# Effect of a multicomponent, person-centred care intervention on client experience and HIV treatment outcomes in Zambia: a stepped-wedge, cluster-randomised trial

**DOI:** 10.1016/S2352-3018(24)00264-9

**Published:** 2024-12-05

**Authors:** Kombatende Sikombe, Aaloke Mody, Charles W Goss, Sandra Simbeza, Laura K Beres, Jake M Pry, Ingrid Eshun-Wilson, Anjali Sharma, Njekwa Mukamba, Lloyd B Mulenga, Brian Rice, Jacob Mutale, Alida Zulu Dube, Musunge Mulabe, James Hargreaves, Carolyn Bolton Moore, Charles B Holmes, Izukanji Sikazwe, Elvin H Geng

**Affiliations:** **Implementation Science Unit, Centre for Infectious Disease Research in Zambia, Lusaka, Zambia** (K Sikombe MPH, S Simbeza MPH, J M Pry PhD, A Sharma PhD, N Mukamba MPH, J Mutale MSc, A Zulu Dube Dip, M Mulabe BSc, C Bolton Moore MBChB, I Sikazwe MBChB); **Department of Public Health Environments and Society, Faculty of Public Health and Policy, London School of Hygiene and Tropical Medicine, London, UK** (K Sikombe, B Rice PhD, Prof J Hargreaves PhD); **Division of Infectious Diseases, Washington University School of Medicine, St Louis, MO, USA** (A Mody MD, C W Goss PhD, Prof E H Geng MD); **Department of International Health, Johns Hopkins University Bloomberg School of Public Health, Baltimore, MD, USA** (L K Beres PhD); **Department of Public Health Sciences, School of Medicine, University of California, Davis, CA, USA** (J M Pry); **Johnson and Johnson, Cape Town, South Africa** (I Eshun-Wilson MD); **Zambian Ministry of Health, Lusaka, Zambia** (Prof Lloyd Mulenga MBChB); **Sheffield Centre for Health and Related Research (SCHARR), School of Medicine and Population Health, University of Sheffield, Sheffield, UK** (B Rice); **Division of Infectious Diseases, University of Alabama, Birmingham, AL, USA** (C Bolton-Moore); **Department of Medicine, Georgetown University, Washington, DC, USA** (Prof C B Holmes MD)

## Abstract

**Background:**

Recipients of health services value not only convenience but also respectful, kind, and helpful providers. To date, research to improve person-centred HIV treatment has focused on making services easier to access (eg, differentiated service delivery) rather than the interpersonal experience of care. We developed and evaluated a Person-Centred Care (PCC) intervention targeting healthcare worker practices.

**Methods:**

Using a stepped-wedge, cluster-randomised design, we randomly allocated 24 HIV clinics stratified by size in Zambia into four groups and introduced a PCC intervention that targeted caring aspects of the behaviour of health-care workers in one group every 6 months. The intervention entailed training and coaching for health-care workers on PCC practices (to capacitate), client experience assessment with feedback to facilities (to create opportunities), and small performance-based incentives (to motivate). In a probability sample of clients who were pre-trained on a client experience exit survey and masked to facility intervention status, we evaluated effects on client experience by use of mean score change and also proportion with poor encounters (≤8 on 12-point survey instrument). We examined effects on missed visits (ie, >30 days late for next scheduled encounter) in all groups and retention in care at 15 months in group 1 and group 4 by use of electronic health records. We assessed effects on treatment success at 15 months (i.e., HIV RNA concentration <400 copies per mL or adjudicated care status) in a prospectively enrolled subset of clients from group 1 and group 4. We estimated treatment effects with mixed-effects logistic regression, adjusting for sex, age, and baseline care status. This trial is registered at the Pan-African Clinical Trials Registry (202101847907585) and is completed.

**Results:**

Between Aug 12, 2019, and Nov 30, 2021, 177 543 unique clients living with HIV made at least one visit to one of the 24 study clinics. The PCC intervention reduced the proportion of poor visits based on exit surveys from 147 (23·3%) of 632 during control periods to 33 (13·3%) of 249 during the first 6 months of intervention, and then to eight (3·5%) of 230 after 6 months (adjusted risk difference [aRD] for control *vs* ≥6 months intervention −16.9 percentage points, 95% CI −24·8 to −8·9 Among all adult scheduled appointments, the PCC intervention reduced the proportion of missed visits from 90 593 (25·3%) of 358 741 during control periods to 40 380 (22·6%) of 178 523 in the first 6 months, and then 52 288 (21·5%) of 243 350 after 6 months (aRD for control *vs* the intervention −4·2 percentage points, 95% CI −4·8 to −3·7). 15-month retention improved from 33 668 (80·2%) of 41 998 in control to 35 959 (83·6%) of 43 005 during intervention (aRD 5·9 percentage points, 95% CI 0·6 to 11·2), with larger effects in clients newly starting treatment (aRD 12·7 percentage points, 1·4 to 23·9). We found no effect on treatment success (based on viral load) in a nested subcohort (379 [83·7%] of 453 in the control phase *vs* 402 [83·8%] of 480 in the intervention phase; aRD 0·9 percentage points, −5·4 to 7·2).

**Interpretation:**

Improving the caring aspects of health-care worker behaviour is feasible in public health settings, enhances client experience, reduces missed appointments and increases retention.

**Funding:**

The Bill and Melinda Gates Foundation.

## Introduction

In public sector settings of health service delivery, prioritising client experience through respectful and friendly interactions might be important, and perhaps necessary, to achieve sustained client retention in HIV treatment programmes.^1^ In the initial decade of the global scale-up of HIV treatment (i.e., approximately 2005–15), programmes emphasised size, scale, and standardisation to rapidly expand infrastructure, workforce, and supply chains, as exemplified by WHO’s 4-S framework.^2^ However, as programmes matured and health and clinical stability of the client population improved, it became clear that the mere presence of services is not enough. To be most effective, services are increasingly seeking to minimise opportunity costs, accommodate client preferences, and offer respectful and caring experiences.^3–5^

Many efforts to improve HIV treatment programmes in high-prevalence settings have focused on novel models, such as differentiated service delivery approaches, which reduce the frequency and decentralise the location of clinical encounters.^1^ However, few innovations emphasise an alternative but important domain of person-centred services: the interpersonal client experience of providers.^1^ In previous qualitative work, we and other authors reported that feeling cared for^6^ was a strong motivator of retention in care, particularly among clients with a low-income status.^7^ Conversely, rude and disrespectful interactions triggered disengagement with health services,^8^ particularly in people with existing challenges, such as psychosocial barriers (eg, stigma and depression).^5^ In a discrete choice experiment in Zambia, a method taken from marketing research, we identified that clients who were lost to follow-up were willing to travel 40 km farther or wait more than 10 h to see a provider who they considered to be kind.^4^

Although the need for kind and person-centred services is clear, some stakeholders, from health-care workers to policy makers, are sceptical that friendly experiences in the public sector are feasible.^9^ Crowded clinics, limited infrastructure (i.e., inadequate facilities, equipment, resources, support systems for health care workers) and demanding hours, exacerbated by major events such as the COVID-19 pandemic, result in burnout and moral injury for health-care workers.^10^ Mental health needs among health-care workers are often not addressed.^11,12^ Yet, surveys also show that health-care workers are motivated by compassion and seek to care, even in public sector settings. In this study, we test the hypothesis that a theory-based, multicomponent strategy comprised of training and facilitation; systematic measurement and feedback of client experience data; and a small incentive to promote respectful, person-centred care (PCC) can improve client experience, retention, and clinical outcomes.^13^ We conducted the study within routine care settings and at a large scale to credibly inform national systems in Zambia and beyond.

## Methods

### Study design

We did a type 2 hybrid, implementation effectiveness trial using a stepped-wedge, cluster-randomised design at 24 public sector facilities in Lusaka province, Zambia, between Aug 12, 2019, and Nov 30, 2021 (over a total of 27 months). Clinics were operated by the Zambian Ministry of Health and supported by the Centre for Infectious Disease Research in Zambia, a Zambian non-governmental organisation. Our study objective was to assess how a multicomponent PCC intervention comprising training and coaching for health-care workers (i.e., practice facilitation), client experience assessment with feedback to health-care workers, and small performance-based incentives could change the behaviour of health-care workers and clients’ experiences, and then ultimately, how these changes affected retention and viral suppression under real-world conditions of service delivery in Zambia. The overall study had four aims, first to evaluate the implementation of the PCC intervention in routine care setting in Zambia; second to evaluate the effect of the PCC intervention on service delivery and client experience; third to evaluate the effect of the PCC intervention on retention and viral suppression; and fourth to evaluate the cost and cost effectiveness of the PCC intervention. In this Article, we present results on client experience, missed visits, retention in care, and viral suppression. Forthcoming analyses will include a mixed-methods evaluation of implementation, changes in client–provider communication and incremental cost-effectiveness of the PCC intervention.

The trial received approval (including a waiver of consent to use electronic health records [EHRs] records) from the institutional review boards (IRB) of the University of Zambia (008–03–19), the University of Alabama at Birmingham (IRB-300003282), and the Zambian Ministry of Health, with multiple US institutions agreeing to rely on using a single IRB with University of Alabama at Birmingham for the review and approval of the study. Participants who were recruited for assessment of client experience and treatment success provided written informed consent. The trial was registered with the Pan African Trial Registry on Jan 29, 2021, under identification number PACTR202101847907585. Registration of the trial was delayed due to an administrative error. There were no major changes to any study procedures or outcomes between initial protocols for institutional review board approval and trial registration (except for 3-month extension and modification of the missed visits analysis in response to the COVID-19 pandemic).

### Participants

We did the study at the cluster level (i.e., health facilities; appendix p 2), with four groups of clinics. The intervention directly targeted healthcare worker behavior at these facilities, and all individuals who accessed care at participating facilities were exposed to either intervention or control conditions during the course of receiving routine clinical care and follow-up at these facilities. In this paper we report four outcomes: client experience, missing the next visit, retention in care, and treatment success. Measurements for each outcome were assessed in distinct cohorts that were independently designed and had independent sample size calculations from within this overall target population (appendix p 2) and are described in more detail below.

### Randomisation and masking

A statistician not otherwise involved in the study randomly assigned clinics into four groups stratified by clinic size and proportion of individuals with a prior viral load test coverage. We allocated eight clinics each to group 1 and group 4 and four each to group 2 and group 3 (appendix p 2) to place more clusters (i.e., an important driver of statistical power) in groups with sufficient continuous periods of exposure in either the treatment or control conditions to enable outcomes that required periods longer than a single step to unfold (e.g., retention at 15 months). We introduced the intervention sequentially to the four groups every 6 months. Due to study interruption caused by the COVID-19 pandemic, period 2 was extended from 6 months to 9 months. Thus, the overall study lasted 27 months over four periods. Investigators and providers were not masked to intervention status, but clients were. Additional details of participant allocation and masking are included in the Procedures section.

### Procedures

The PCC intervention was a multicomponent approach targeting the behaviour of health-care workers based on formative work emphasising the importance of friendliness, respect, dignity, adequate communication, and involving clients in decision making. We organised these ideas into a theory of change^4,5,14–16^ using the PRECEDE-PROCEED model (which advocates for interventions that predispose, enable, and reinforce behaviour change) and Michie and colleagues’ capability, opportunity, and motivation segmentation of mechanisms of behaviour change (appendix p 3).^17,18^ We developed the intervention protocol through a week-long, human-centred design workshop in 2018 with 20 health-care workers (appendix pp 4, 46).^13^

The Intervention comprised three components (appendix pp 3–6). First, we delivered a 2-day interactive, off-site training programme to build knowledge and skills for person-centred care, targeting all clinic staff in HIV treatment and maternal–child health units. The training was led by experienced healthcare workers (i.e. nurses) who had worked in the region before. Content focused on communication, stress management, teamwork, and person-centred principles (eg, empathy and shared decision making). Training was followed by weekly to monthly mentorship visits to facilitate translation of concepts into practical steps in day-to-day care. Second, we introduced client exit surveys, using an adapted, validated, 12-item survey instrument. The results were presented at quarterly staff meetings, during which data from clinical outcomes (e.g. viral load suppression) were reviewed. Third, we provided biannual clinic-level incentives up to US$75 for clinics with the best or most improved client experience metrics. (appendix pp 4–6).

### Outcomes and Measurements

To understand the extent to which our intervention was delivered and implemented in routine care settings, we documented the occurrence of each of the three components of the intervention. We documented the extent to which we measured client experience and outcomes as intended; the success of training, data sharing, and coaching; and the process of implementing the incentive.

To evaluate client experience, we trained selected clients (i.e., cohort 1) on an instrument adapted from the Physician-Patient Communication Behaviour Scale ^19^ that captured key features of care experience—including satisfaction with services, HCW attitude, and communication. The Trained Exit Client approach (TEC) is a variation of standardised client methods.^20^ Across all periods at the 16 clinics in groups 2, 3, and 4, we recruited a systematic sample of clients (every nth client, where n depended on the clinic size) on the days of their visits but before entering the facility. Recruitment was stratified by clients either currently in care or those returning to care after being more than 30 days late for an appointment. Individuals who were aged 18 or older, attending a visit for HIV care that day at a clinic in groups 2, 3, or 4, and able to recall events, comprehend instructions and pass a literacy assessment were eligible for the TEC approach. Exclusion criteria included current or previous staff at the health facility, pregnant, and being acutely ill. Participants underwent a 40–60 min single one-on-one training session in private.^20^ These procedures aimed to establish clear standards for the visits and promote client attentiveness, thereby minimising recall and social desirability bias. Clients then presented for their routine visits and the survey was administered immediately after. Providers were unaware of which clients were trained, and trained clients were masked to clinic intervention status. Trained clients served for one encounter and were excluded from the treatment success sub cohort, and otherwise received routine care.

We assessed retention in two ways: missing a visit by more than 30 days and retention at 15 months. These outcomes used data from the Zambian national EHR for people living with HIV, which includes data on all individuals receiving HIV care in Zambia. First, we assessed the proportion of visits for which clients missed their next scheduled appointment by more than 30 days to assess short-term effects of our intervention on retention (i.e. cohort 2) (appendix p2). For assessment of missed next visits, we used EHRs to identify visits from all individuals aged 18 or older receiving care at all 24 facilities across all groups and periods (ie, cohort 2) (appendix p2). These outcomes were assessed at the visit level for all visits where the next scheduled appointment was at least 30 days before database closure and missed visits could be determined across all groups and study periods. Second, all clients aged 18 or older who visited group 1 or group 4 clinics during period 1 were assessed for retention in care at 15 month using data from the national EHR (ie, cohort 3) (appendix p2). Retention at 15 months was defined as having at least one visit between 11 months and 19 months after the initial visit in period 1 (i.e., time zero). We restricted to group 1 (intervention) and group 4 (control) clinics to ensure that individuals had at least 15 months of uninterrupted exposure to intervention or control conditions without crossover before outcome determination (appendix p 2).

The treatment success cohort (i.e., a composite indicator of viral suppression, cohort 4) was a nested sample enrolled during period 1 (i.e., the first six months) in group 1 (i.e., intervention) and group 4 (i.e., control) clinics and then followed for 15 months (i.e., period 3) (appendix p2). This allowed for sufficient continuous observation time in either treatment or control conditions. Treatment success was defined as plasma HIV RNA concentration of less than 400 copies per mL regardless of care status or documented evidence of being in care and on ART in the absence of a viral load measurement. During period 1, we recruited a systematic sample (i.e., every nth client, where n depended on the clinic size) of adults aged 18 years or older making an HIV care visit at a group 1 (i.e. intervention) or group 4 (i.e. control) clinics. Exclusion criteria included being aged under 18, pregnant and unable or willing to provide consent. Recruitment was stratified to ensure sufficient numbers for precise estimates in each of 3 subgroups: 1) new antiretroviral therapy (ART) starters, 2) individuals already on ART and in care, and 3) individuals who were returning to clinic after being more than 30 days late for an appointment (returners).

Individuals in the treatment success cohort, who were unaware of the intervention status, received routine clinical care without any direct interactions or monitoring by the study team until outcome ascertainment at the 15-month endpoint (within a window of +4 months or −4 months; appendix p 2). We used routinely collected viral load data when present and either facilitated sample collection during routine care visits or outreach (via phone and in-person tracing) to obtain viral load data and additional care history (eg, deaths, transfers or travel, ART possession, and missed visits) from clients, providers, and informants in the community. For clients with missing viral load data after these steps, we did an extended outcome and investigation classification adjudication process, where five masked adjudicators independently reviewed all available data (eg, previous viral loads outside the outcome window but during the study period, missed visits, transfers, and other care history obtained during tracing attempts) using a standard algorithm (appendix pp 7–8). In cases where adjudicators disagreed, consensus was reached by discussion. Outcome ascertainment was originally planned at 12 months but was extended to 15 months due to COVID-19 interruptions. All viral load samples (ie, routine and study collected) were run on the Cobas CAP/CTM assay (Roche Molecular Systems, Branchburg, NJ, USA).

We used national EHR data to augment data on other client characteristics for all four cohorts EHR data is collected by health care workers during routine visits and contains sociodemographic (eg, age, sex, marital status, education, and clinic site; ethnicity data were not collected), clinical (eg, HIV viral loads, enrolment CD4 counts, and WHO stage), encounter (eg, HIV clinic enrolment date, ART initiation date, and follow-up visits), and facility-level characteristics (eg, size). Adverse effects attributed to the intervention were not recorded.

### Statistical analysis

Our study sought to show effects at a level of scale credible to regional, national, and international health systems, and we therefore sought to carry out the intervention at the maximal scale possible under logistical and financial constraints. For client experience (ie, cohort 1), we assumed that 65% of individuals reported a positive client experience at baseline, anticipated that a 15 percentage point risk difference was meaningful, and assumed an intraclass correlation of 0·2, with which we achieve 80% power and at p<0·05 with 15–30 surveys per clinic per period in groups 2, 3, and 4. Due to pragmatic constraints on sample size, we excluded clinics a priori in group 1 from TEC measurements on the basis of power calculations that suggested there was statistical efficiency in restricting the available sample to groups 2, 3, and 4, where both within-clinic and between-clinic comparisons are possible (appendix p 2). Stratified analyses were prespecified but not necessarily powered to detect differences.^23^ Sample size for cohort 2 (ie, visits from all individuals with one or more encounters at all 24 facilities during the entire study period) and for cohort 3 (ie, all individuals making a visit in the 16 clinics in group 1 and group 4 in period 1) were determined by practical and not statistical considerations. Given that making visits could be ascertained from the clinical EHR, we leveraged all available data in the EHR and identified the bounds of detectable effects given the numbers available. For the outcome of treatment success (in cohort 4, the systematic sample in who we enrolled to assess treatment success at 15 months), we assumed that 75% of individuals were virally suppressed at baseline and a conservative intraclass correlation between clinics of 0·2 based on previous epidemiological research by our group.^23^ Based on the fixed number of clinics, we estimated that we would be able to detect a 10·7 percentage point difference a priori between intervention and control groups, with 80% power and at p<0·05 if we recruited approximately 60 clients at each clinic in group 1 and group 4, for a total maximum of approximately 960 (480 in control and 480 in intervention) viral loads.

In cohort 1 (ie, the sample recruited to assess client experience in groups 2, 3, and 4), we first used mixed-effects linear regression to assess effects on the sum score of the 12-item client experience instrument. Exposure to the PCC intervention was categorised as control, early intervention (ie, <6 months), and late intervention (ie, ≥6 months) to account for time to see maximal effects. Period was treated as a fixed-effect to adjust for secular trends based on standard analysis of stepped-wedge designs.^21^ We adjusted for baseline characteristics (as described for the primary analysis) both for overall and stratified analyses.^21^ In an adjunctive analysis, we dichotomised scores as above or below a threshold of ≤8. (ie, approximately the 15th percentile) and used mixed-effects logistic regression to compare the risk of having a bad clinic experience (ie, sum score ≤8) among the intervention groups and report the number needed to treat. We used post-estimation commands to transform estimates into the number of clients needed to be exposed to a facility that underwent the PCC intervention to avoid a bad clinic experience, akin to the number needed to treat. Finally, we used quantile regression analysis to explore variation of effects by baseline experience.

In cohort 2 (i.e., all visits in 24 clinics), we used mixed-effects logistic regression with a categorical exposure to the PCC intervention (i.e., control, early intervention, or late intervention) and period as a fixed effect^21^ on the outcome of being more than 30 days late for an appointment. As some individuals might have had more than one visit, we accounted for clustering at both the individual level and the clinic level using random effects. Each visit was categorised as control or intervention based on when it occurred (e.g., an individual might have one visit assigned as control and a later visit categorised as intervention after the clinic crossed over from control to intervention). We did a sensitivity analysis also examining the time to a missed visit using a Kaplan–Meier approach (which was specified in our initial protocol but modified due to COVID-19 in the final statistical analysis plan). For this analysis, time zero was the first visit in the control or intervention period (individuals could contribute person time to both periods), and individuals were censored at the time of crossover from control to intervention, database closure, or at the time of the first missed visit.

In cohort 3 (i.e., assessed on all clients who visited group 1 or group 4 clinics during period 1), we used mixed-effects logistic regression to examine the effect of the PCC intervention on retention in care at 15 months in period 3, adjusting for care status at baseline (i.e., newly starting ART, in care, returning to care after being >30 days late [i.e., returners]), sex, age, and time previously in care to enhance precision and reduce bias.^22^ We stratified analyses by care status (i.e., in care, new ART, or returners), sex, and age (i.e., <25, 25–44, and ≥45 years). We also conducted a sensitivity analysis restricted to individuals enrolled in cohort 4.

Lastly, in cohort 4 (i.e., subsample enumerated from group 1 and group 4 clinics during period 1), we used mixed-effects logistic regression to assess the effect of the PCC intervention on treatment success at 15 months in period 3, using a similar analytic approach as for cohort 3 but adjusting for baseline viral suppression. We also conducted a sensitivity analysis that did not incorporate adjudicated outcomes, where missing HIV RNA measurements were treated as treatment failures.

For all analyses, we report estimates as raw absolute risk and means, and adjusted risk differences and mean differences under treatment versus control conditions, using post-estimation commands in Stata (version 17.0) or SAS (version 9.4) for conversions as needed. For adjusted analyses, individuals with missing covariate data were excluded (figure). All analyses were prespecified and conducted according to the statistical analysis plan developed and finalised a priori (appendix p 25–36).

The study did not have an independent data safety monitoring board; however, a study advisory board met to review study progress and provide ongoing advice around study implementation.

### Role of the funding source

The funder of the study had no role in study design, data collection, data analysis, data interpretation, or writing of the report.

## Results

Between Aug 12, 2019, and Nov 30, 2021, 177 543 unique clients living with HIV made at least one visit to one of the 24 study clinics (figure). In cohort 1 (ie, a sample of individuals who were either in care or returning to care after being >30 days late for an appointment), we assessed 1420 clients for eligibility and enrolled 1165 (82·0%) clients (table 1). 1111 (78·2%) clients answered all questions on the study instrument. Cohort 2 comprised 780 614 visits in the EHR made by clients across all facilities and periods, in whom we assessed whether the next visit was missed by more than 30 days. Characteristics across all study cohorts were balanced by treatment condition. In cohort 3 (i.e., individuals who made any visit in period 1 in group 1 and group 4), we identified 85 003 individuals who were eligible for outcome assessment 15 months later. Lastly, in cohort 4 (i.e., treatment success), we assessed 1226 clients for eligibility and enrolled 933 (76·1%) clients from 16 clinics (figure; table 1). 447 (47·9%) of 933 were in care (395 [90·6%] suppressed of the 436 with a baseline viral load ), 262 (28·1%) were newly initiating on ART, and 224 (24·0%) were returners previously on ART (124 [56·6%] suppressed of the 219 with a baseline viral load ).

We provided training to 2567 health-care workers (913 [90.1%] of 1013 health-care workers in ART and 1654 [88.9%] of 1861 health-care workers in maternal and child health and outpatient departments) and ancillary staff (e.g., security guards and cleaners) across all clinics. Of 3961 health-care workers employed across all facilities, we trained nurses (883 [59·4%] of 1486), counsellors (841 [66·1%] of 1272), general workers (153 [77·3%] of 198), community health workers (65 [80·2%] of 81), and medical doctors (35 [40·7%] of 86; appendix p 9). Mentorship visits occurred at a median of 3 per clinic per month (IQR 2–4; 1234 total mentorship visits), but frequencies did vary over time as mentors tailored the frequency of visits up or down as needed (eg, in response to COVID-19; appendix pp 10–11). Across all facilities during intervention periods, we collected 2488 routine exit survey measurements (mean 41·5 per clinic per period, SD 5.4) and successfully conducted data review meetings every 3 months as planned (120 in total) to feedback client experience data and prioritise indicators (appendix p 12). All facility-level incentives were delivered as intended on a biannual basis.

The PCC intervention led to improved client experience in the first 6 months, with larger effects observed after 6 months of the intervention (table 2). Although the intervention improved client experience overall, the greatest effect was seen in reducing negative experiences (ie, sum score ≤8 on a 12-point survey instrument): the proportion of participants with a negative experience decreased from 23.3% (147/632) during control periods to 13·3% (33/249) during the first six months of intervention, and then to 3·5% (8/230) after 6 months (table 2; appendix p 13). The number needed to be exposed to a facility that underwent the PCC intervention to avert a bad experience was 5·9 (95% CI 4·0 to 11·2; appendix pp 14–15). Quantile regression also displayed larger reductions among clients at the lowest end of the client experience distribution compared to those with better experiences. For example, after 6 months of intervention, we observed a 3·0-point (95% CI 1·1 to 4·9) improvement for those with client experiences at the 5th quantile, 1·3-point (0·3 to 2·3) improvement at the 25th quantile, and 0·6-point (−0·1 to 1·3) improvement at the median, but we did not observe any improvement for clients above the 60th quantile (appendix pp 13, 16).

In cohort 2 (ie, all visits across all facilities during the entire study period), we identified that the PCC intervention reduced the probability of a missed visit (ie, >30 days late) from 25·3% (90,593/358,741) during control periods to 22·6% (40,380 /178,523) in the first 6 months, and then to 21·5% (52,288/243,350) after 6 months (table 3). Results were consistent using a time-to-event approach (appendix pp 17–18).

In cohort 3 (all clients with an encounter during period 1 in the 16 clinics in group 1 and group 4), we identified that retention in care at 15 months improved by 5·9% (95% CI: 0·6-11·2%) during intervention (table 4; appendix p 19). We observed slightly larger effects among new ART starters and individuals re-engaging in care compared with those in care at baseline (p< 0·0001 for interaction). These patterns across subgroups were similar to those seen with missing the next visit. We also saw slightly larger effects in younger age groups than older age groups (p< 0·0001 for interaction) and a non-significant difference among women compared with men (p=0·20 for interaction). Overall results were also generally similar when restricted to individuals enrolled in cohort 4, although CIs were wide (appendix p 20).

We obtained HIV RNA measurements for 783 clients (380 [83·9%] of 453 in control *vs* 403 [84·0%] of 480 in intervention phases) in cohort 1, and classified treatment outcomes in the remaining 150 (16·1%) of 933 clients using the extended outcome and investigation classification algorithm that incorporated all available evidence on care engagement, including information obtained in extensive attempts to trace the client (appendix p 21). There were no differences in deaths or official transfers between groups (appendix p 22). We observed no effect of the intervention on treatment success at 15 months (table 5). We identified no difference in most subgroups, but intervention effects on treatment success appeared to be higher in clients younger than 25 years compared with those who were older (p=0·18 for interaction). A sensitivity analysis in which missing HIV RNA measurements were treated as failures showed similar results, although the intervention effect was greater in clients younger than 25 years old (appendix p 23).

## Discussion

At present, person-centred care in health is broadly endorsed but encompasses a wide range of concepts. Scholl and colleagues, for example, identify 15 domains from taking a biosocial perspective to improving access (e.g., reducing waiting times).^24^ Although all are worthwhile in principle, advancing the science of person-centred services should involve testing specific practices that can feasibly be used to improve outcomes. Most studies of person-centred care have focused on the aspects of making care easier by use of differentiated service delivery models (eg, increasing access via fast-track medication pick-up).^1^ By contrast, this study explores improving the care experience for clients by making care nicer, through coaching providers to greet, communicate, and work with clients to improve rapport and relationships. This study implies that an approach focused on the provider–client interpersonal relationship represents a distinct but viable public health strategy that can complement other well-known mechanisms, such as increasing access.

The effects of the PCC strategy were most pronounced in reducing the poorest client experiences as shown in the quantile regression analyses and generally in populations that are traditionally not well engaged, such as new ART and returning clients (based on sub-group analyses), findings that have implications for reducing disparities. After 6 months of the PCC intervention, the number of visits that were scored 8 or less on a 12-point survey instrument reduced from 147 (23·3%) of 632 to eight (3·5%) of 230, an approximately 85% reduction. The study intervention also seemed to have larger effects on 15-month retention for clients newly starting treatment, those returning after missing appointments, and younger individuals. Larger effects in these groups, who experience greater stigma and are navigating the implications of HIV for relationships and livelihoods, support the hypothesis that a human touch is most meaningful at particularly precarious moments. A PCC approach that improves provider–client relationships offers a counterpoint to other person-centred approaches, such as differentiated service delivery that focuses on making care smoother for stably suppressed clients already doing well.^1^

In 1980, Lipsky’s widely influential theory of the street-level bureaucrat observed that government and service workers need to exercise discretion when interacting with the public.^25^ Our training and coaching sought not only to improve providers communication (e.g., greeting clients well) but also to make providers aware of the discretion that they have as the interface between public health and the public, and to use it, when possible, to support client care. Although our quantitative data were not designed to capture this effect, some qualitative interviews provided clues and will be reported in forthcoming papers. In one example, a health-care worker noted “There are times when a client scheduled for drug pick up later comes to the facility and says, ‘I have come to pick medicines, I am going far away’. A health care worker would then shout at a client saying ‘…your date is not today…how can you come today?’ But with PCC, we came to understand that you are not supposed to shout at a client [and] …understand why he has come early and then you help them….”.

One important contribution of this study is to close the knowledge gap about how to implement person-centred care by offering a package that can be feasibly reproduced in real-world health system settings. Both the 2020 *Lancet Global Health* Commission on high-quality health systems and WHO advocate client-centred, kind, and caring services^26,27^ but do not provide concrete models for promoting person-centred care delivery. The training and facilitation approach that we used mirrored the existing site-support function routinely provided in Zambia by the Ministry of Health or non-governmental organisations, such as the Centre for Infectious Disease Research in Zambia.^28^ We designed and deployed a measurement system for client experience and showed how to incorporate these insights into existing staff data-review meetings. These approaches can also synergise and integrate with existing efforts to strengthen community-led monitoring.^29^ In short, the study offers a practical roadmap and proposes key metrics for translating emerging policy priorities around person-centred care into practice, even beyond HIV care.^30–33^

Limitations of the study included shortcomings of the stepped-wedge design. Although motivated by a perceived absence of equipoise, stepped-wedge designs also limit comparisons of effects that require time to reach full strength, since fewer randomised units undergo the longest periods of treatment than short periods of treatment. Because some of our most important outcomes (ie, 15-month retention and treatment success) require time for the effect to be seen, additional effects could have been observed under a parallel cluster design. Second, the study was disrupted by the COVID-19 pandemic, which might have influenced findings from period 2 onwards. However, the fact that the study continued with a relatively minimal extension of 3 months even during the pandemic is a testament to the flexibility of the approach. Third, analyses for our outcome of viral suppression were, in retrospect, likely underpowered because of an overly optimistic 10% absolute difference in sample size calculations. Finally, although the trained clients were not aware of clinic intervention status, our study TEC trainers were aware of clinic intervention status and could have introduced interviewer bias.

In short, we studied an intervention designed collaboratively with health-care workers that leverages health-care workers’ innate desire to deliver PCC but manage constraints under which they operate.^13^ We showed in more than 175 000 clients in 24 clinics located in Zambia that training, ongoing health-care worker support, and data on client experience helped to change day-to-day practice, influenced the experience of clients, improved retention, and changed quality of care. Our results suggest that providing public health services in a way that is friendly and warm is not inimical to services at scale and might have large impacts over time: the study itself was done in 24 clinics that treat 90% of all clients in Lusaka province and, considering the size of exposed population and estimates of numbers needed to treat, the study results would translate to approximately 50 000 visits with a bad experience, 14 000 missed visits, and 6200 episodes of loss to follow-up averted. Prioritisation of the public health care experience represents a complementary pathway to improve services in HIV care and beyond (eg, in maternal–child health), where disrespectful interactions are a barrier to effective care.^30–32^ Future work should consider integration of these strategies and client experience metrics into national quality improvement plans.

## Supplementary Material

Supplementary material

## Figures and Tables

**Figure 1:**
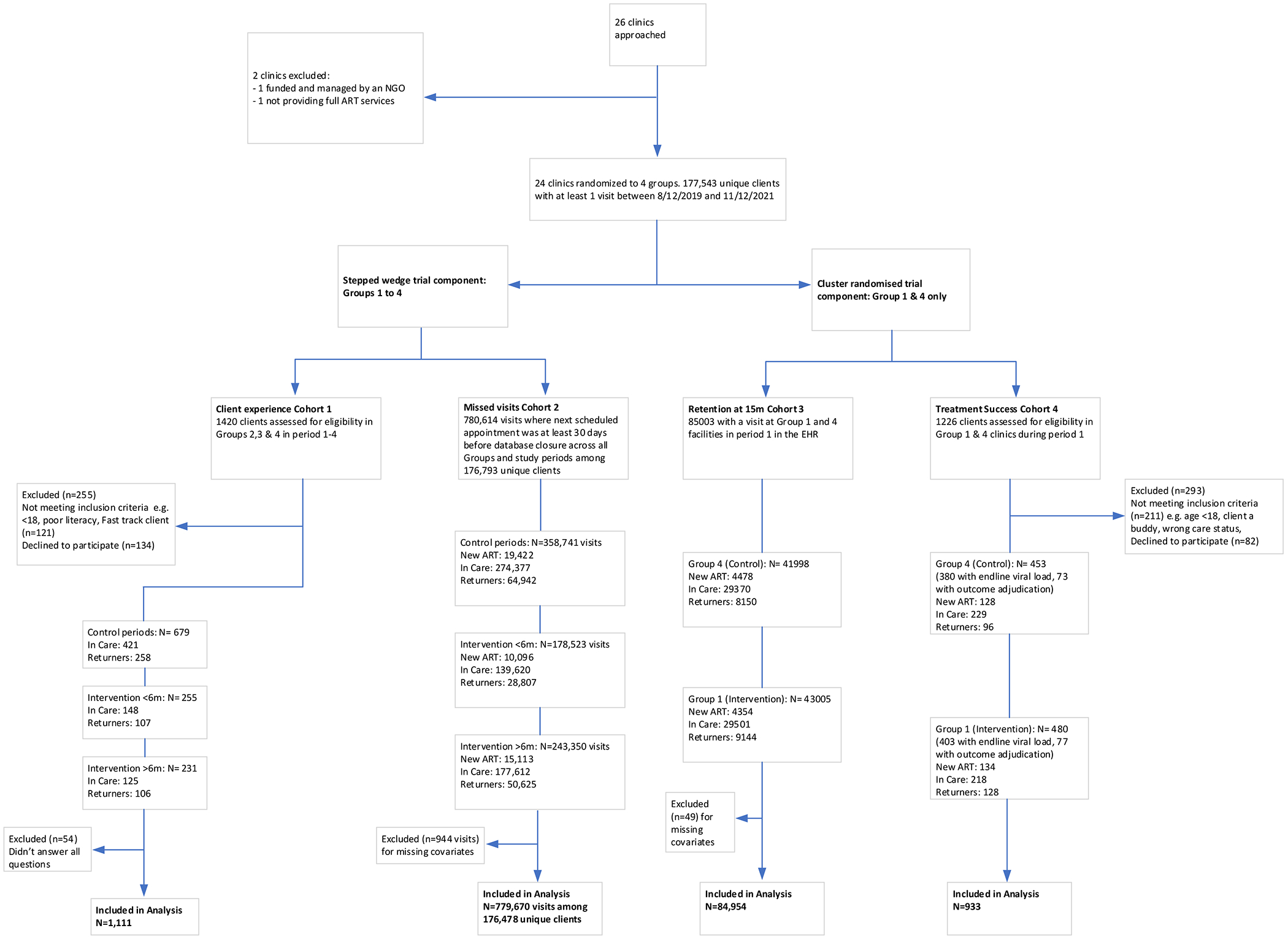
Flow diagram of inclusion criteria for analysis of treatment success, retention, client experience, and missed visits All cohorts were derived from the 177 543 clients who made at least one visit during the study period at one of the 24 clinics and were at some point exposed to either intervention or control periods (or both). Treatment success and client experience cohorts were actively enrolled, whereas cohorts for retention in care and missed visits were derived from the EHRs. Client experience (ie, measured in facilities in groups 2, 3, and 4) and missed visits (ie, measured in all groups) were cross-sectional outcomes and were assessed with a stepped-wedge, cluster-randomised design, where intervention exposure was categorised into three-levels: control, intervention duration of less than 6 months, and intervention duration of more than 6 months. Retention in care and treatment success at 15 months (ie, longitudinal outcomes) were assessed among individuals at group 1 and group 4 clinics only, in what amounts to a parallel cluster-randomised design. EHR=electronic health record. ART=antiretroviral therapy.

**Table 1:** Participant characteristics for patient experience, missed visits, retention in care, and treatment success

	Cohort 1: client experience (n=1165)			Cohort 2: 30 days late before next visit* (n=176 793 unique individuals; 780 614 visits)			Cohort 3: in care at 15 months (n=85 003)		Cohort 4: treatment success (n=933)	
	Control (n=679)	<6 months intervention (n=255)	≥6 months intervention (n=231)	Control (n=107 220 unique individuals; 358 741 visits)	<6 months intervention (n=111 429 unique individuals; 178 523 visits)	≥6 months intervention (n=90 984 unique individuals; 243 350 visits)	Control (n=41 998)	Intervention (n=43 005)	Control (n=453)	Intervention (n=480)
Sex										
Female	337 (49·6%)	130 (51·0%)	120 (51·9%)	227 172 (63·3%)	112 240 (62·9%)	151 429 (62·2%)	27 461 (65·4%)	27 530 (64·0%)	266 (58·7%)	273 (56·9%)
Male	342 (50·4%)	125 (49·0%)	111 (48·1%)	131 569 (36·7%)	66 283 (37·1%)	91 921 (37·8%)	14 537 (34·6%)	15 475 (36·0%)	187 (41·3%)	207 (43·1%)
Age at study enrolment^†^, years	38 (31–46)	38 (31–45)	37 (31–45)	39 (32–46)	39 (31–46)	38 (31–45)	39 (32–46)	39 (32–46)	37 (31–44)	37 (30–44)
Age group at study enrolment, years										
<25	57 (8·4%)	28 (11·0%)	24 (10·4%)	29 122 (8·1%)	15 307 (8·6%)	23 353 (9·6%)	3183 (7·6%)	3073 (7·1%)	46 (10·2%)	47 (9·8%)
25–34	216 (31·8%)	84 (32·9%)	77 (33·3%)	100 401 (28·0%)	50 609 (28·3%)	72 088 (29·6%)	11 744 (28·0%)	11 876 (27·6%)	146 (32·2%)	152 (31·7%)
35–44	233 (34·3%)	86 (33·7%)	83 (35·9%)	131 923 (36·8%)	64 346 (36·0%)	86 160 (35·4%)	15 701 (37·4%)	15 916 (37·0%)	161 (35·5%)	171 (35·6%)
45–59	155 (22·8%)	52 (20·4%)	45 (19·5%)	85 414 (23·8%)	41 928 (23·5%)	53 888 (22·1%)	10 026 (23·9%)	10 621 (24·7%)	92 (20·3%)	107 (22·3%)
>60	18 (2·7%)	5 (2·0%)	2 (0·9%)	11 561 (3·2%)	6123 (3·4%)	7447 (3·1%)	1321 (3·1%)	1493 (3·5%)	8 (1·8%)	3 (0·6%)
Missing	0	0	0	320 (0·1%)	210 (0·1%)	414 (0·2%)	23 (0·1%)	26 (0·1%)	0	0
Group										
Group 1	··	··	··	0	73 285 (41·1%)	179 423 (73·7%)	··	43 005 (100·0%)	··	480 (100·0%)
Group 2	110 (16·2%)	81 (31·8%)	164 (71·0%)	36 956 (10·3%)	41 399 (23·2%)	42 449 (17·4%)	··	··	··	··
Group 3	120 (17·7%)	58 (22·7%)	62 (26·8%)	110 311 (30·7%)	36 654 (20·5%)	21 478 (8·8%)	··	··	··	··
Group 4	449 (66·1%)	116 (45·5%)	5 (2·2%)	211 474 (58·9%)	27 185 (15·2%)	0	41 998 (100·0%)	··	453 (100·0%)	··
Period										
Period 1	290 (42·7%)	··	··	163 209 (45·5%)	73 285 (41·1%)	0	41 998 (100·0%)	43 005 (100·0%)	453 (100·0%)	480 (100·0%)
Period 2	177 (26·1%)	81 (31·8%)	43 (18·6%)	141 437 (39·4%)	41 399 (23·2%)	86 485 (35·5%)	··	··	··	··
Period 3	212 (31·2%)	58 (22·7%)	65 (28·1%)	54 095 (15·1%)	36 654 (20·5%)	86 435 (35·5%)	··	··	··	··
Period 4	0	116 (45·5%)	123 (53·2%)	0	27 185 (15·2%)	70 430 (28·9%)	··	··	··	··
Care status										
New ART	··	··	··	19 422 (5·4%)	10 096 (5·7%)	15 113 (6·2%)	4478 (10·7%)	4353 (10·1%)	128 (28·3%)	134 (27·9%)
In care	421 (62·0%)	148 (58·0%)	125 (54·1%)	274 377 (76·5%)	139 620 (78·2%)	177 612 (73·0%)	29 370 (69·9%)	29 511 (68·6%)	229 (50·6%)	218 (45·4%)
Returner	258 (38·0%)	107 (42·0%)	106 (45·9%)	64 942 (18·1%)	28 807 (16·1%)	50 625 (20·8%)	8150 (19·4%)	9141 (21·3%)	96 (21·2%)	128 (26·7%)
Viral suppression at study enrolment										
Suppressed	··	··	··	··	··	··	··	··	260 (57·4%)	261 (54·4%)
Not suppressed	··	··	··	··	··	··	··	··	187 (41·3%)	209 (43·5%)
Missing	··	··	··	··	··	··	··	··	6 (1·3%)	10 (2·1%)
WHO stage at ART enrolment										
Stage 1	332 (48·9%)	128 (50·2%)	106 (45·9%)	183 346 (51·1%)	90 625 (50·8%)	124 407 (51·1%)	21 434 (51·0%)	21 746 (50·6%)	241 (53·2%)	256 (53·3%)
Stage 2	87 (12·8%)	30 (11·8%)	31 (13·4%)	47 081 (13·1%)	22 856 (12·8%)	29 202 (12·0%)	5423 (12·9%)	5560 (12·9%)	71 (15·7%)	46 (9·6%)
Stage 3	94 (13·8%)	40 (15·7%)	33 (14·3%)	57 761 (16·1%)	28 700 (16·1%)	36 250 (14·9%)	6815 (16·2%)	7020 (16·3%)	50 (11·0%)	49 (10·2%)
Stage 4	7 (1·0%)	0	1 (0·4%)	4270 (1·2%)	2507 (1·4%)	3246 (1·3%)	500 (1·2%)	664 (1·5%)	2 (0·4%)	3 (0·6%)
Missing	159 (23·4%)	57 (22·4%)	60 (26·0%)	66 283 (18·5%)	33 835 (19·0%)	50 245 (20·6%)	7826 (18·6%)	8015 (18·6%)	89 (19·6%)	126 (26·3%)
Date of HIV care enrolment										
<2010	101 (14·9%)	34 (13·3%)	24 (10·4%)	65 127 (18·2%)	31 146 (17·4%)	36 778 (15·1%)	7569 (18·0%)	7531 (17·5%)	58 (12·8%)	47 (9·8%)
2010–2014	95 (14·0%)	34 (13·3%)	32 (13·9%)	53 268 (14·8%)	25 924 (14·5%)	33 013 (13·6%)	6902 (16·4%)	7141 (16·6%)	58 (12·8%)	48 (10·0%)
2014–2017	135 (19·9%)	46 (18·0%)	40 (17·3%)	64 332 (17·9%)	31 028 (17·4%)	39 308 (16·2%)	8225 (19·6%)	8503 (19·8%)	73 (16·1%)	78 (16.3%)
2017–2019	195 (28·7%)	61 (23·9%)	64 (27·7%)	76 009 (21·2%)	37 655 (21·1%)	49 141 (20·2%)	10 201 (24·3%)	11 037 (25·7%)	92 (20·3%)	120 (25·0%)
>2019	153 (22·5%)	80 (31·4%)	71 (30·7%)	100 005 (27·9%)	52 770 (29·6%)	85 110 (35·0%)	9101 (21·7%)	8793 (20·4%)	172 (38·0%)	187 (39·0%)
Marital status										
Single	91 (13·4%)	40 (15·7%)	37 (16·0%)	43 547 (12·1%)	20 835 (11·7%)	26 868 (11·0%)	4827 (11·5%)	3900 (9·1%)	70 (15·5%)	49 (10·2%)
Married	355 (52·3%)	121 (47·5%)	113 (48·9%)	195 487 (54·5%)	95 590 (53·5%)	131 557 (54·1%)	23 113 (55·0%)	23 954 (55·7%)	257 (56·7%)	234 (48·8%)
Divorced	63 (9·3%)	25 (9·8%)	28 (12·1%)	39 716 (11·1%)	19 340 (10·8%)	26 236 (10·8%)	4609 (11·0%)	4472 (10·4%)	49 (10·8%)	66 (13·8%)
Widowed	48 (7·1%)	20 (7·8%)	14 (6·1%)	29 029 (8·1%)	14 274 (8·0%)	18 816 (7·7%)	3482 (8·3%)	3641 (8·5%)	26 (5·7%)	26 (5·4%)
Missing	122 (18·0%)	49 (19·2%)	39 (16·9%)	50 962 (14·2%)	28 484 (16·0%)	39 873 (16·4%)	5967 (14·2%)	7038 (16·4%)	51 (11·3%)	105 (21·9%)
Education status at ART enrolment										
None	22 (3·2%)	8 (3·1%)	7 (3·0%)	19 361 (5·4%)	9135 (5·1%)	11 467 (4·7%)	2108 (5·0%)	2106 (4·9%)	23 (5·1%)	19 (4·0%)
Primary	98 (14·4%)	32 (12·5%)	37 (16·0%)	92 602 (25·8%)	45 857 (25·7%)	61 282 (25·2%)	10 753 (25·6%)	11 812 (27·5%)	114 (25·2%)	116 (24·2%)
Secondary	384 (56·6%)	152 (59·6%)	128 (55·4%)	174 279 (48·6%)	87 138 (48·8%)	121 241 (49·8%)	20 297 (48·3%)	20 790 (48·3%)	233 (51·4%)	228 (47·5%)
University	61 (9·0%)	20 (7·8%)	26 (11·3%)	20 010 (5·6%)	10 668 (6·0%)	14 184 (5·8%)	1939 (4·6%)	1856 (4·3%)	19 (4·2%)	16 (3·3%)
Missing	114 (16·8%)	43 (16·9%)	33 (14·3%)	52 489 (14·6%)	25 725 (14·4%)	35 176 (14·5%)	6901 (16·4%)	6441 (15·0%)	64 (14·1%)	101 (21·0%)

Data are n (%) or median (IQR). Viral load and retention are longitudinal outcomes that were assessed after 15 months of intervention experience. The variable group 1 is composed of all individuals who were in clinics in which the intervention was rolled out earliest in calendar time. ART=antiretroviral therapy.

* Numbers for this cohort are reported at the visit level. Unique individuals may have visits during both control and intervention periods.

† Numbers of participants who were missing age data are reported in categorical age variables.

**Table 2:** Effect of the PCC intervention on client experience, cohort 1

	Participants	Control (n=632)		<6 months intervention (n=249)		≥6 months intervention (n=230)		Control *vs* <6 months intervention		Control *vs* ≥6 months intervention	
		Sum score*	Bad experience^†^	Sum score*	Bad experience^†^	Sum score*	Bad experience^†^	Adjusted difference for sum score (95% CI; p value)	Adjusted difference for bad experience, percentage points (95% CI; p value)	Adjusted difference for sum score (95% CI; p value)	Adjusted difference for bad experience, percentage points (95% CI; p value)
Overall	1111	9·9 (2·3)	147/632 (23·3%)	10·7 (1·9)	33/249 (13·3%)	11·1 (1·2)	8/230 (3·5%)	0·4 (0·0 to 0·8; 0·036)	−5·4 (−13·4 to 2·6; 0·19)	1·0 (0·5 to 1·5; <0·001)	−16·9 (−24·8 to −8·9; <0·001)
Baseline care status											
In care	651	9·8 (2·4)	98/383 (25·6%)	10·6 (2·1)	21/144 (14·6%)	11 (1·3)	4/124 (3·2%)	0·5 (0·0 to 1·0; 0·057)	−8·0 (−18·4 to −2·5; 0·14)	1·3 (0·6 to 2·0; <0·001)	−20·4 (−30·1 to −10·8; <0·001)
Returner	460	10·1 (2·3)	49/249 (19·7%)	10·9 (1·7)	12/105 (11·4%)	11·1 (1·1)	4/106 (3·8%)	0·3 (−0·3 to 0·9; 0·29)	−2·2 (−13·8 to 9·3; 0·71)	0·6 (−0·2 to 1·4; 0·14)	−11·2 (−22·4 to 0·0; 0·088)
Sex											
Female	566	9·6 (2·4)	8/3198 (27·6%)	10·5 (2·1)	22/127 (17·3%)	11·0 (1·3)	2/120 (1·7%)	0·3 (−0·3 to 0·9; 0·28)	−6·7 (−18·9 to 5·6; 0·29)	1·0 (0·2 to 1·8; 0·010)	−24·0 (−34·8 to −13·2; <0·001)
Male	545	10·2 (2·2)	59/313 (18·8%)	10·9 (1·6)	11/122 (9·0%)	11·2 (1·1)	6/110 (5·5%)	0·5 (−0·1 to 1·0; 0·087)	−5·4 (−15·1 to 4·3; 0·29)	0·8 (0·1 to 1·5; 0·022)	−9·2 (−19·4 to 1·1; 0·12)
Age, years											
<25	95	10·0 (2·1)	8/48 (16·7%)	9·9 (2·7)	6/25 (24·0%)	11·2 (1·1)	0/22	−0·7 (−1·9 to 0·5; 0·24)	28·5 (−2·5 to 59·4; 0·082)	−0·1 (−1·5 to 1·3; 0·89)	−20·2‡
25–44	732	9·7 (2·5)	115/417 (27·6%)	10·6 (2·0)	25/163 (15·3%)	11·0 (1·4)	8/152 (5·3%)	0·3 (−0·2 to 0·9; 0·20)	−6·0 (−16·4 to 4·4; 0·26)	0·8 (0·1 to 1·5; 0·020)	−17·8 (−27·9 to −7·7; 0·002)
>45	284	10·5 (1·9)	24/167 (14·4%)	11·3 (1·1)	2/61 (3·3%)	11·3 (0·8)	0/56	0·8 (0·2 to 1·5; 0·008)	−15·2 (−29·9 to −0·4; 0·10)	1·2 (0·4 to 2·0; 0·002)	−19·3‡

Data are n, mean (SD), or n (%), unless otherwise stated.

* Sum score of 12-item patient experience instrument. Results represent crude mean sum score with standard deviation and adjusted mean differences from mixed-effects linear regression.

† Bad experiences indicate interactions with sum score ≤8 (approximately in bottom 15th percentile). Results represent crude number and percent and adjusted risk differences from mixed-effects logistic regression.

‡ 95% CI and p value were not estimable in regression models due to positivity violations (ie, perfect prediction due to no reported bad patient experiences for intervention ≥6 months).

**Table 3:** Effect of the PCC intervention on missed visits by 30 days, cohort 2

	Visits	Missed visits in the control periods (n=358 741)	Missed visits during <6 months intervention (n=178 523)	Missed visits during ≥6 months intervention (n=243 350)	Control *vs* <6 months intervention		Control *vs* ≥6 months intervention	
					Adjusted risk difference, percentage points (95% CI)	p value	Adjusted risk difference, percentage points (95% CI)	p value
Overall	780 614	90 593/358 741 (25·3%)	40 380/178 523 (22·6%)	52 288/243 350 (21·5%)	−2·1 (−2·5 to −1·7)	<0·001	−4·2 (−4·8 to −3·7)	<0·001
Baseline care status								
New ART	44 631	6715/19 422 (34·6%)	3219/10 096 (31·9%)	4323/15 113 (28·6%)	−1·3 (−2·8 to 0·3)	0·13	−5·0 (−7·4 to −2·7)	<0·001
In care	591 609	63 167/274 377 (23·0%)	28 635/139 620 (20·5%)	33 305/177 612 (18·8%)	−2·5 (−3·0 to −2·1)	<0·001	−4·6 (−5·2 to −3·9)	<0·001
Returner	144 374	20 711/64 942 (31·9%)	8526/28 807 (29·6%)	14 660/50 625 (29·0%)	−0·6 (−1·6 to 0·4)	0·23	−2·1 (−3·6 to −0·6)	0·005
Sex								
Female	490 841	58 508/227 172 (25·8%)	25 733/112 240 (22·9%)	33 001/151 429 (21·8%)	−2·9 (−3·4 to −2·4)	<0·001	−5·6 (−6·3 to −4·9)	<0·001
Male	289 773	32 085/131 569 (24·4%)	14 647/66 283 (22·1%)	19 287/91 921 (21·0%)	−0·7 (−1·3 to −0·1)	0·031	−1·9 (−2·9 to −1·0)	<0·001
Age group, years								
<25	67 782	8628/29 122 (29·6%)	3980/15 307 (26·0%)	6083/23 353 (26·1%)	−2·2 (−3·6 to −0·8)	0·002	−3·1 (−5·1 to −1·0)	0·003
25–44	505 527	60 289/232 324 (26·0%)	26 641/114 955 (23·2%)	34 899/158 248 (22·1%)	−2·2 (−2·6 to −1·7)	<0·001	−4·4 (−5·1 to −3·7)	<0·001
>45	206 361	21 569/96 975(22·2%)	9697/48 051 (20·2%)	11 166/61 335 (18·2%)	−1·9 (−2·6 to −1·1)	<0·001	−4·4 (−5·4 to −3·3)	<0·001

Data are n or n (%), unless otherwise stated. Missed visits are measured among 176 793 unique individuals.

**Table 4:** Effect of the PCC intervention on retention in care at 15 months, cohort 3

	Participants	In Care at 15 months, Control (n=41 998)	In Care at 15 months, Intervention (n=43 005)	Adjusted risk difference, (95% CI)	p value
Overall	85 003	33 668/41 998 (80·2%)	35 959/43 005 (83·6%)	5·9 (0·6 to 11·2)	0·026
Baseline care status					
New ART	8831	1980/4478 (44·2%)	2447/4353 (56·2%)	12·7 (1·4 to 23·9)	0·030
In care	58 881	25 123/29 370 (85·5%)	25 727/29 511 (87·2%)	4·1 (0·2 to 8·0)	0·038
Returner	17 291	6565/8150 (80·6%)	7785/9141 (85·2%)	5·0 (0·1 to 10·1)	0·049
Sex					
Female	54 991	21 805/27 461 (79·4%)	22 872/27 530 (83·1%)	6·6 (0·8 to 12·4)	0·022
Male	30 012	11 863/14 537 (81·6%)	13 087/15 475 (84·6%)	3·7 (0·0 to 7·4)	0·051
Age group, years					
<25	6256	1994/3183 (62·7%)	2111/3073 (68·7%)	7·5 (−1·0 to 16·0)	0·087
25–44	55 237	21 574/27 445 (78·6%)	23 031/27 792 (82·9%)	6·9 (1·1 to 12·7)	0·018
>45	23 461	10 088/11 347 (88·9%)	10 804/12 114 (89·2%)	1·2 (−1·3 to 3·7)	0·34

Data are n or n (%).

**Table 5:** Effect of the PCC intervention on treatment success at 15 months, cohort 4

		Treatment Success, Control (n=453)	Treatment Success, Intervention (n=480)	Adjusted risk difference, (95% CI)	p value
Overall	933	379/453 (83·7%)	402/480 (83·8%)	0·9 (−5·4 to 7·2)	0·78
Baseline care status					
New ART	262	99/128 (77·3%)	106/134 (79·1%)	2·1 (−13·8 to 18·1)	0·79
In care	447	211/229 (92·1%)	199/218 (91·3%)	−0·8 (−7·2 to 5·5)	0·80
Returner	224	69/96 (71·9%)	97/128 (75·8%)	3·4 (−10·5 to 17·2)	0·63
Baseline treatment success status					
Plasma HIV RNA suppressed	519	239/260 (91·9%)	240/261 (92·0%)	0·0 (−6·9 to 6·8)	0·99
Plasma HIV RNA not suppressed	396	136/187 (72·7%)	152/209 (72·7%)	0·5 (−10·7 to 11·7)	0·93
Sex					
Female	539	224/266 (84·2%)	237/273 (86·8%)	2·6 (−5·9 to 11·0)	0·55
Male	394	155/187 (82·9%)	165/207 (79·7%)	−1·3 (−10·0 to 7·4)	0·77
Age group, years					
<25	92	32/46 (69·6%)	38/47 (80·9%)	13·6 (−1·4 to 28·6)	0·11
25–44	630	261/307 (85·0%)	269/323 (83·3%)	−1·1 (−7·0 to 4·9)	0·72
>45	210	86/100 (86·0%)	95/110 (86·4%)	2·1 (−5·3 to 9·5)	0·65

## Data Availability

The Government of Zambia allows data sharing when applicable local conditions are satisfied. In this case, the data from the study will be made available to any interested researchers on request. Study data are de-identified and archived in a secure locally maintained server. The Centre for Infectious Disease Research in Zambia Ethics and Compliance Committee is responsible for approving such requests. Requests for data access should be made to the Secretary to the Committee/Head of Research Operations, Mrs Hope Chinganya (Hope.Chinganya@cidrz.org), mentioning the intended use for the data. The committee will then facilitate review and authorisation to release the data as requested. Data requests must include contact information, a research project title, and a description of the intended use.

## Research in context

### Evidence before this study

We did a systematic review (registered at PROSPERO, CRD42020203639) to identify interventions to improved person centred care focused on health-care worker and client interactions to identify their effects on HIV-related outcomes in low-income and middle-income countries. Our latest search, Oct 1, 2023, included Embase 1947-, MEDLINE 1946-, Cochrane Library, Web of Science, American Psychological Association PsycInfo, Cumulative Index to Nursing and Allied Health Literature Plus 1937-, and Global Health and abstracts of major HIV conferences in recent years (2001-2021) We used the Cochrane and Newcastle-Ottawa Scale risk of bias tools and evaluated certainty of evidence using the Grading of Recommendations, Assessment, Development, and Evaluations framework. Among the 28 studies included with a comparison arm and available effect sizes, 10 of the 16 (62·5%) with HIV-care-continuum outcomes and 28 of the 28 with client-reported outcomes (100·0%) reported a significant positive effect on at least one the outcomes, respectively. No meta-analysis was done due to heterogeneity of the strategies examined. We identified three cluster-randomised trials, two of which were in specific populations (ie, adolescents and female sex workers). The one previous study in a general adult population did not measure provider behaviour or client experience as an outcome. Additionally, many studies of improved client experience are focused on models of care, such as differentiated service delivery models, that alter location and frequency of encounters rather than on the interpersonal (ie, health-care worker–client) experience.

### Added value of this study

The study shows the potential effectiveness of a multicomponent strategy co-designed with front-line health-care workers. By using synergistic mechanisms of building capacity (ie, external practice facilitation), creating opportunity (ie, introduction of an audit of client experience), and creating motivation (ie, a small facility-level performance incentive) for health system and health-care worker behaviour, this research provides robust experimental data on improving person-centred practices in health systems. The study was conducted in 24 government-operated facilities, and the findings argue that person-centred services can be feasibly implemented under routine service delivery conditions in Zambia and similar settings. Importantly, the study emphasises the crucial role of client reporting in driving improvements in health-care worker behaviour. The study showed a significant improvement in client experience (with 5.9 individuals needing to be exposed to avoid a poor experience), and modest but meaningful effects on retention, with larger effects among new ART starters. The study did not observe an effect on treatment success, based on measurement of viral load. These results extend the understanding of how targeted strategies can improve the quality of person-centred care in low- and middle-income settings.

### Implications of all the available evidence

Existing research suggests that disrespectful, non-person-centred care drives clients away from health services, and this study moves the science of person-centred care forwards by rigorously testing and showing a tangible and reproducible intervention. The key intervention mechanisms (ie, training and support to refresh health-care workers on pre-service training in person-centred care, integrating client experience data, and introducing a small facility-level reputational incentive) provide readily adaptable principles that can serve as a basis for other health conditions, including maternal–child services, where advancing health-care quality, client experience, and dignity might also be needed. Although there are many dimensions to person-centred services, we suggest that focusing on a specific dimension(ie, the interpersonal relationship between health-care workers and clients to improve client experience)can effectively complement common approaches focused on the architecture of HIV services (eg, community drug-distribution points and other differentiated service delivery models). Health-care worker behaviour should also be considered an underemphasised but potentially major area for improvement. For future research, the findings emphasise the need to explore the application of these person-centred principles across different health conditions and settings. Finally, friendly services have been studied mostly in youth or other key populations, and this study emphasises the notion that client-centred services and experience are both feasible and appropriate in a general adult population, suggesting that strategies could be beneficially extended to broader public health settings.
